# Supplementary material for: Polyketide synthase gene clusters in the ericoid mycorrhizal fungus Oidiodendron maius: genome-wide prediction, expression profiling, and functional characterization of knockout mutants
Source: Front Plant Sci. 2026 Sep 16;17:1924430. doi: 10.3389/fpls.2026.1924430 (PMC13623903; doi:10.3389/fpls.2026.1924430)
Supplement: Supplementary file 1 [file SupplementaryFile1.zip › Supplementary Materials/Supplementary Folder 1.zip!/Oidma1_AssemblyScaffolds_Repeatmasked/knownclusterblast/cluster45/jgi.p_Oidma1_60484_mibig_hits.html]

| MIBiG Protein | Description | MIBiG Cluster | MiBiG Product | % ID | % Coverage | BLAST Score | E-value |
| --- | --- | --- | --- | --- | --- | --- | --- |
| BAD29973.1 | transporter\_protein | BGC0000676 | Aphidicolin\_biosynthetic\_gene\_cluster | 35 | 75.4385964912 | 275 | 1.2e-73 |
| ACD39756.1 | major\_facilitator\_superfamily\_transporter | BGC0000076 | Hypothemycin\_biosynthetic\_gene\_cluster | 27 | 75.4385964912 | 193 | 1.1e-48 |
| ACD39765.1 | major\_facilitator\_superfamily\_transporter | BGC0000077 | Hypothemycin\_biosynthetic\_gene\_cluster | 27 | 75.4385964912 | 193 | 1.1e-48 |
| ACP19369.1 | SaqJ1 | BGC0000267 | Saquayamycin\_Z\_/\_galtamycin\_B\_biosynthetic\_gene\_cluster | 28 | 63.8888888889 | 146 | 1.5e-34 |
| ADG86335.1 | transporter | BGC0000190 | A-74528\_biosynthetic\_gene\_cluster | 27 | 63.1578947368 | 144 | 4.3e-34 |
| AAL15595.1 | Sim17 | BGC0000270 | Simocyclinone\_biosynthetic\_gene\_cluster | 28 | 61.2573099415 | 138 | 2.4e-32 |
| AGO50605.1 | transporter | BGC0000229 | Grincamycin\_biosynthetic\_gene\_cluster | 28 | 59.9415204678 | 138 | 3.1e-32 |
| AAK06799.1 | putative\_export\_protein\_SimEx | BGC0001072 | Simocyclinone\_biosynthetic\_gene\_cluster | 27 | 61.2573099415 | 136 | 1.2e-31 |
| ABC87523.1 | putative\_drug\_efflux\_transporter | BGC0001011 | Meridamycin\_biosynthetic\_gene\_cluster | 27 | 60.3801169591 | 126 | 1.6e-28 |
| AMY15055.1 | MFS\_transporter | BGC0001339 | Squalestatin\_S1\_biosynthetic\_gene\_cluster | 24 | 65.7894736842 | 125 | 2.1e-28 |
